# Gross lesions associated with *Sarcocystis miescheriana* in a wild boar hunted for human consumption: the importance of trained hunters to ensure animal health surveillance and food safety

**DOI:** 10.1007/s00436-024-08342-7

**Published:** 2024-09-10

**Authors:** Madalena Vieira-Pinto, Francesco Chiesa, Isabel Cristina Ribeiro Pires, Carmen Gonzalez Duarte, Selene Rubiola

**Affiliations:** 1 Department of Veterinary Sciences, Universidade de Trás-os-Montes e Alto Douro (UTAD), 5000-801 Vila Real, Portugal; 2 Veterinary Research Centre and Associate Laboratory for Animal and Veterinary Sciences (AL4AnimalS), UTAD, Vila Real, Portugal; 3 Department of Veterinary Sciences, University of Turin, Largo Paolo Braccini 2, 10095 Grugliasco, TO Italy; 4 Clube Português de Monteiros, Alcabideche, Portugal

**Keywords:** *Sarcocystis* spp., Game meat, Wild boar, *Sarcocystis suihominis*, *Sarcocystis miescheriana*

## Abstract

*Sarcocystis* is a genus of protozoa with a worldwide distribution infecting a wide range of animals, including humans. Wild boars can harbor at least two species of *Sarcocystis*, that is, the zoonotic *Sarcocystis suihominis*, using humans as definitive hosts, and *Sarcocystis miescheriana*, for which wild and domestic canids serve as definitive hosts. In Portugal, hunting holds significant economic and social importance, and wild boars are among the most appreciated hunted species. As the consumption of wild boar meat can expose humans to several foodborne pathogens, the presence of trained hunters can make a difference in ensuring animal health surveillance and food safety. Herein, we report the detection of macroscopic cystic lesions associated with *S. miescheriana* in a wild boar hunted for human consumption, resulting in carcass condemnation. To the best of the authors’ knowledge, the presence of *S. miescheriana* in wild boar tissues has never been associated with macroscopic pathological alterations before. Although *S. miescheriana* cannot infect humans, carcasses affected by grossly visible pathological changes must be declared unfit for consumption. Therefore, our finding points out the potential economic damage associated with carcass rejection due to the presence of gross lesions associated with generalized sarcocystosis. Nonetheless, further studies are required to investigate these alterations that currently appear to be occasional findings.

## Introduction

The genus *Sarcocystis* includes more than 220 species of intracellular protozoan parasites with a worldwide distribution (Dubey et al. 2016). *Sarcocystis* spp. can affect a wide range of mammals, birds, reptiles, and possibly fishes, which are part of a two-host predator-prey life cycle. Carnivores and omnivores can act as definitive hosts by the ingestion of muscle tissue of the intermediate hosts containing sarcocysts, while herbivores and omnivores can act as intermediate hosts by ingesting food or water contaminated by oocysts or sporocysts shed by the definitive hosts through the feces. Among wild game species, wild boars (*Sus scrofa*) can serve as definitive hosts for at least two *Sarcocystis* spp.: *Sarcocystis miescheriana* (Kuhn 1865; Labbé 1899), which uses canids as definitive hosts, and the zoonotic *Sarcocystis suihominis* (Tadros and Laarman 1979; Heydorn 1977), for which humans and non-human primates serve as definitive hosts. The existence of a third cat-transmitted species using wild and domestic swine as intermediate hosts, named *Sarcocystis porcifelis* in 1976, has never been confirmed (Dubey 1976).

Humans can become infected by consuming raw or undercooked pork, thereby developing intestinal sarcocystosis, a foodborne illness characterized by gastrointestinal symptoms including nausea, vomiting, abdominal pain, and diarrhea. Symptoms can vary in intensity depending on the number of ingested sarcocysts; nevertheless, natural infections often result in asymptomatic or mildly symptomatic manifestations, thereby going unnoticed (Rosenthal 2021). Wild and domestic pigs, including wild boars, usually develop subclinical infections, although weight loss, purpura, muscle tremors, dyspnoea, abortion, and even death have been described in experimental infections (Dubey et al. 2016). In 2011, Caspari et al. reported the first case of naturally acquired fatal sarcocystosis associated with severe myocarditis, hepatitis, and nephritis in a domestic pig (Caspari et al. 2011).

*S. suihominis* and *S. miescheriana* do not usually cause visible lesions or alterations observable at slaughter. Indeed, while cattle *Sarcocystis* spp. are frequently associated with bovine eosinophilic myositis, a myopathy causing carcass condemnation or devaluation and economic losses for farmers (Dubey and Rosenthal 2022; Rubiola et al. 2024), the presence of inflammatory reactions associated with *S. miescheriana* or *S. suihominis* has been occasionally reported in boar tissues (Calero-Bernal et al. 2015; Gazzonis et al. 2019; Pacifico et al. 2023), causing only histologically detectable alterations. According to Regulation (EU) 2019/627, any carcass infected with parasites or containing macroscopically visible lesions, such as eosinophilic myositis or cystic alterations, should be declared unfit for human consumption and condemned, totally or partially. Actually, only three documented cases of macroscopic sarcocystosis leading to carcass discard in swines have been reported, characterized by whitish cystic lesions, up to 8 mm long, disseminated through most of the skeletal muscle (Rubiola et al. 2023; Chikweto et al. 2024; Obadiah et al. 2024).

In Portugal, hunting holds significant economic and social importance, and wild boars are among the most appreciated hunted species (Vieira-Pinto et al. 2014). Those engaged in hunting, along with their families and friends, can face the risk of contracting foodborne parasites, including *S. suihominis*, if they consume undercooked or raw wild boar meat such as dry-cured homemade sausages, as previously reported (Vieira-Pinto et al. 2021). In this context, the presence of trained hunters, promoted by Regulation (EC) No. 853/2004, can make a difference in ensuring animal health surveillance and food safety. Nevertheless, training programs aiming at improving food safety along the game meat chain are not actually harmonized throughout Europe. For this purpose, the University of Trás-os-Montes e Alto Douro (UTAD) in Portugal offers training courses approved by the National Veterinary Authority and accredited by UTAD’s Scientific Council to undertake an initial examination of wild game onsite. The 16-hour courses for large game enable hunters to become trained persons, covering the subjects dictated in Regulation (EC) No. 853/2004. These courses are organized in collaboration with hunting associations, and, so far, 29 courses have trained over 450 hunters nationwide.

Hereafter, we report for the first time the presence of macroscopic cystic lesions, detected by a trained hunter, associated with S. *miescheriana* in a wild boar hunted for human consumption, resulting in carcass destruction.

## Materials and methods

In May 2023, after hunting a wild boar (male, adult) in the Alentejo region of Portugal, a trained hunter began eviscerating and deboning the animal and noticed small whitish spots on various thigh muscles. The abnormal muscle pattern raised concerns, prompting the hunter to photograph the lesions and contact the national hunter training coordinator for advice. After consulting the national hunter training coordinator, a portion of muscle was taken and preserved in formaldehyde, while a subset was frozen for further molecular analysis. The remaining muscles were not examined. Although the wild boar was in good condition, the hunter properly disposed of the entrails and the carcass.

The muscle tissue fixed in 10% buffered formalin was stored at room temperature and processed by routine procedures for histopathological examination: samples were embedded in paraffin wax, processed to create 4-µm-thick longitudinal and transverse sections, and stained with hematoxylin and eosin (H/E).

Likewise, five of the macroscopic, whitish, oval cyst-like lesions were isolated from the surrounding muscle tissue, excised, and individually stored at −20 °C for molecular examination. Additionally, a 10 g aliquot of healthy skeletal muscle tissue was used to perform the direct microscopic examination as described by Moré et al. (2011), in order to check for the presence of microscopic cysts. Muscle tissue was blended in a tissue homogenizer with the addition of up to 50 ml of phosphate-buffered saline (PBS); the resulting muscle homogenate was centrifuged, and the pellet was resuspended in 20 ml of PBS and observed using an inverted microscope at × 40 magnification (Nikon TMS). Detected cysts or cyst portions were individually collected and frozen at −20 °C for molecular identification.

All the grossly detected cystic lesions and the microscopic cysts or cyst portions observed using the inverted microscope were individually submitted to DNA extraction using the animal tissues protocol of the DNeasy Blood and Tissue® Kit (Qiagen, Hilden, Germany). Each sample was tested for the presence of *Sarcocystis* spp. DNA through the amplification of the partial cytochrome C oxidase subunit I mitochondrial (mtDNA *cox1*) gene (~1100 bp) using the primer set SF1-SR11 (Gazzonis et al. 2019) as described by Rubiola et al. (2023). Moreover, DNA extracted from the macroscopic whitish cyst-like structures was tested for the presence of Taeniidae DNA using the primer pair JB3–JB4.5, targeting a fragment of the mtDNA *cox1* gene, in order to evaluate the potential involvement of porcine cysticercosis in the occurrence of the grossly visible alterations (Bowles et al. 1992).

The resulting amplicons were purified and sequenced with ExoSAP-IT™ Express (Thermo Fisher Scientific, USA) and the BigDye™ Terminator version 3.1 Cycle Sequencing Kit (Applied Biosystems, Thermo Fisher Scientific, USA). Sequencing products were purified with a NucleoSEQ kit (Macherey-Nagel, Düren, Germany); 10 µl of each purified product were mixed with 10 µl of formamide and sequenced on an Applied Biosystems SeqStudio Genetic Analyzer (Thermo Fisher Scientific, Waltham, MA). Sequencing results were analyzed by manually assembling forward and reverse sequences into consensus sequences using the Alignment Explorer feature of MEGA 11 (Kumar et al. 2016). Sequence similarity searches in GenBank were done using the nucleotide BLAST algorithm at the NCBI database. Multiple alignments were obtained by using the ClustalW algorithm in MEGA 11. Sequences were truncated at both ends, so that all partial sequences started and ended at the same nucleotide positions. Phylogenetic analyses were conducted using the Neighbor-Joining algorithm (test of phylogeny: bootstrap method, 1000 replications; substitution model: p-distance model) within MEGA 11 on 69 partial sequences from 57 taxa, including 8 new sequences generated in the present study, a *Neospora caninum* and an *Eimeria tenella* sequences used as outgroups and 59 *Sarcocystis* spp. sequences retrieved from GenBank. Accession numbers of the sequences included in the analyses are shown within the phylogenetic tree.

## Results

**Fig. 1:**
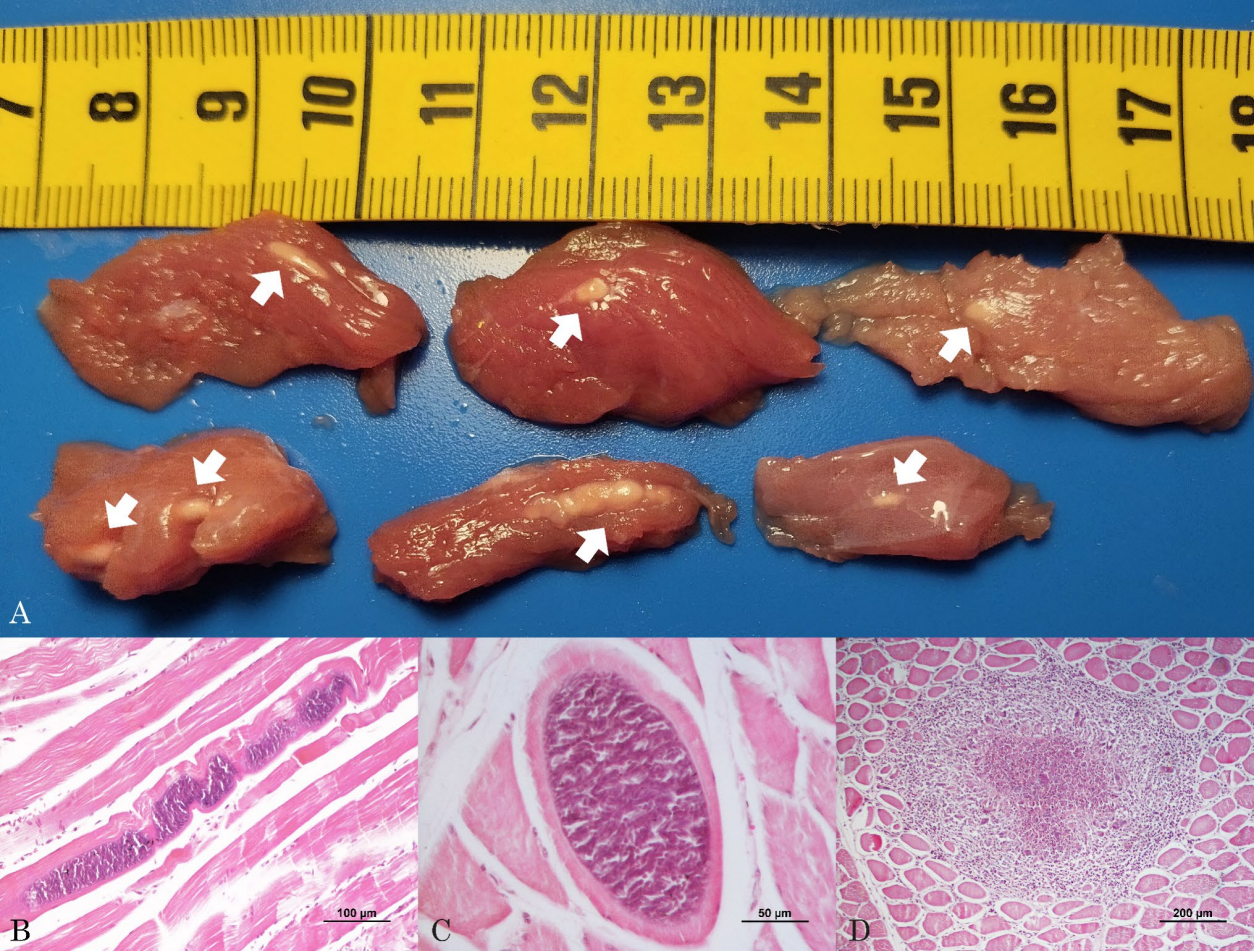
**A** White cystic alterations detected in the leg muscles of the condemned wild boar carcass. **B**, **C** *Sarcocystis* spp. cysts detected in muscles of the condemned wild boar carcass stained with hematoxylin and eosin. **D** Muscular granuloma with necrosis and inflammatory infiltrate compatible with the rupture of one of the parasitic cysts

At gross examination, several white, firm cystic structures, up to 13 mm in length, similar to rice grains, were identified in the leg muscles of the wild boar carcass (Fig. 1A). At histology, cytoplasmatic basophilic structures compatible with *Sarcocystis* spp. cysts were found in the analyzed muscle tissue. The cysts were round, oval, or elongated (Fig. 1B, C) depending on the cutting plane and measured 80–130 µm × 80–243 µm in size. The cyst wall measured up to 3.4 µm in thickness, and palisade-like protrusions were observed in most of them. Additionally, multiple granulomas of varying sizes were observed (Fig. 1D). Necrosis extension varied among the granulomas, while eosinophilic infiltration was consistent in all cases. The inflammatory infiltrate also included macrophages and multinucleated giant cells, which were compatible with the rupture of parasitic cysts. However, no obvious cyst wall-like material was observed in any of the granulomas. Eosinophilic amorphous material among the cellular debris was also observed in the center of the granulomas, with some lightly basophilic material. Unfortunately, no gross cyst-like lesions were included in the portion of muscle collected and preserved in formaldehyde for the histopathological examination.

The macroscopic cystic lesions collected for molecular identification (*n* = 5) tested negative for the presence of Taeniidae DNA; on the contrary, all the lesions tested positive for the presence of *Sarcocystis* DNA using primer pair SF1-SR11. Sanger sequencing of the products resulted in 1039–1053 bp sequences showing identity of 94–99.6% with *Sarcocystis miescheriana* GenBank entries (MH404185–MH404227; MT070614–MT070635; OQ472068–OQ472077; LC349977–LC349980; OR859843–OR859862) (best match, *S. miescheriana*) and less than 79.5–79.9% with *S. suihominis* or any other *Sarcocystis* species.

**Fig. 2:**
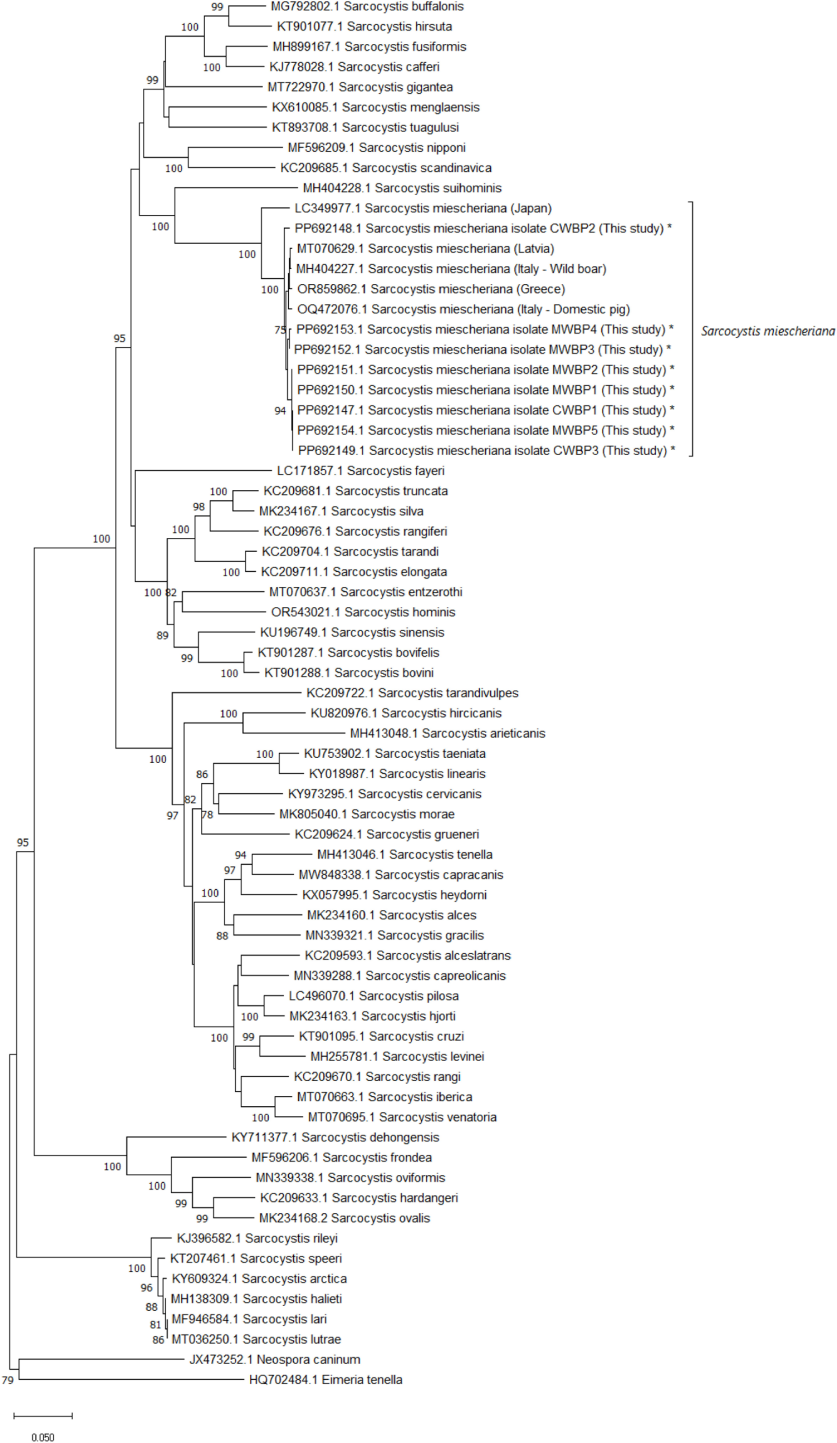
Phylogenetic tree inferred using the Neighbor-Joining method, based on 974 nucleotide positions of the mtDNA *cox1* gene of 69 sequences from 57 taxa, including 8 sequences generated in the present study (marked with an asterisk) (1000 replications). The evolutionary distances were computed using the *p*-distance method. Branch support values lower than 75 are not shown next to the branches. A sequence of *Neospora caninum* and a sequence of *Eimeria tenella* were used as outgroups

The microscopic examination allowed the detection of three thick-walled cysts and cyst portions in the healthy muscle tissue. All the tested microscopic cysts (*n* = 3) resulted positive for the presence of *Sarcocystis* DNA; sanger sequencing of the amplicons resulted in 984–1045 bp sequences with a percentage of identity of 93.9–99.5% with *Sarcocystis miescheriana* GenBank entries (MH404185–MH404227; MT070614–MT070635; OQ472068–OQ472077; LC349977–LC349980; OR859843–OR859862) (best match, *S. miescheriana*), and less than 79.6–81.1% identity with *S. suihominis* or any other *Sarcocystis* sp. The phylogenetic analysis was based on 974 nucleotide positions of the mtDNA *cox1* gene and showed a close relationship between the *S. miescheriana* sequences generated in the present study and the *S. miescheriana* sequences generated in Italy, Latvia, and Greece, while a higher genetic distance separated all the abovementioned sequences from the *S. miescheriana* sequences generated in Japan (Fig. 2). Sequences generated in the present study have been uploaded to the GenBank database under accession numbers PP692147–PP692154.

## Discussion and conclusions

The present study reports for the first time the presence of gross cystic lesions associated with *S. miescheriana* in a wild boar hunted for human consumption, leading to carcass discard. Recently, three cases of macroscopic sarcocystosis have been documented in pig carcasses condemned at slaughter (Rubiola et al. 2023; Chikweto et al. 2024; Obadiah et al. 2024), thereby increasing the interest in this parasitosis; however, to the best of the authors’ knowledge, the presence of *S. miescheriana* in wild boar tissues has never been associated with macroscopic alterations before.

Data on the occurrence of *Sarcocystis* spp. in wild boars in Portugal are limited to two studies conducted around 10 years ago (Coelho et al. 2014, 2015), indicating the absence of the zoonotic *S. suihominis* and a *S. miescheriana* infection rate of 37–39% and 74%, respectively. These results highlight, on one hand, a medium to high level of exposure to the canid-transmitted *S. mieschieriana* and, on the other hand, a low zoonotic risk for the consumer of wild boar meat in Portugal. Trained hunters and hunting dogs might play a role in the life cycle of *S. miescherian*a, and in the surveillance of foodborne diseases and occasional pathological findings such as the one reported herein (Basso et al. 2020). Although *S. miescheriana* cannot infect humans and poses no direct risk to consumers of raw or undercooked meat, according to the Regulation (EU) 2019/627, carcasses affected by grossly visible pathological changes must be declared unfit for human consumption (Commission Implementing Regulation and (EU) 2019/627 (2019)). Therefore, our finding points out the potential economic damage associated with carcass rejection due to the presence of gross lesions associated with generalized sarcocystosis. Nonetheless, further studies are required to investigate these alterations that currently appear to be occasional findings.

The molecular characterization here performed has allowed us to rule out the possible role of other parasites which might be associated with degenerated cystic lesions, such as calcified cysticerci. The phylogenetic analysis based on the mtDNA *cox1* gene showed close relationships between the *S. miescheriana* sequences generated in the present study and the *S. miescheriana* specimens isolated in wild boars in Latvia, Greece, and Italy; a greater phylogenetic distance could be noticed between our sequences and the sequences generated from the *S. miescheriana* specimens isolated in wild boars in Japan, thereby showing a relation between the genetic similarity of the samples and their geographical distance (Prakas et al. 2020). While the histological examination here performed has allowed the identification of both intact sarcocysts and granulomas, the collected material was not enough to enable further morphological investigations, such as scanning electron microscopy (SEM) and transmission electron microscopy (TEM), which could give a better description of the host abnormal reaction to the presence of *S. miescheriana* cysts. Further research should be performed to better investigate these unusual pathological alterations.

To control diseases in wild game, early detection and timely mitigation measures are crucial. Hunters, who have frequent contact with animals and the surrounding territory, play a key role in safeguarding the health of these populations. Proper training for hunters, as outlined in Regulation (EU) 853/2004, is essential for this purpose. This training should help raise awareness of risk factors and control measures for the transmission of infectious agents, enabling hunters to implement effective mitigation measures. A well-trained hunter is essential for monitoring diseases, implementing good hygiene practices, and protecting game handlers. Furthermore, hunters play a crucial role in promoting good disease control practices, such as the proper disposal of by-products, making them a key component of the One Health approach in the hunting sector. For this purpose, hunters must receive training to the satisfaction of the competent authority to become proficient; hunting organizations should be actively encouraged to participate in these training courses. Training programs on good hunting practices must include topics such as the recognition of abnormal behaviors and pathological changes in wild game caused by diseases, together with hygiene rules; moreover, hunters have to master proper techniques for handling, transporting, and eviscerating wild game animals. Presently, a COST Action aiming at harmonizing methodologies and establishing specific guidelines and training courses for safe game meat production in Europe is ongoing (https://www.cost.eu/actions/CA22166/).

In conclusion, here we reported the first case of macroscopic cystic lesions associated with *S. miescheriana* in wild boars, resulting in carcass condemnation. Further studies are needed to update our knowledge on the prevalence of *Sarcocystis* spp. in wild boars in Portugal, including the zoonotic *S. suihominis*, and to monitor these unusual pathological findings, thereby ensuring animal health surveillance and food safety.

## Data Availability

Sequences generated in the present study have been uploaded to the GenBank database under accession numbers PP692147–PP692154.
